# Prof. Miller’s Work on Human Parturition

**Published:** 1849-11

**Authors:** 


### PROFESSOR MILLER'S WORK ON HUMAN PARTURITION.

We are happy to find that the favorable opinion we expressed of this book, is abundantly confirmed by the "American Journal of Medical Sciences." The last number of that leading American medical periodical gives a very commendatory review of the book, and does justice to its merits. B.

